# Tobacco Poisoning

**Published:** 1883-03

**Authors:** J. W. Thomson

**Affiliations:** Springfield, Mass.


### TOBACCO POISONING.

J. W. Thomson, M. D., Springfield, Mass.

On March 12th, 1878, Mr. A. S., æt. 40, called at my office. He was a broad-shouldered, powerful man, six feet one and a half inches without his boots; eyesight failing, which he attributed to the inordinate use of tobacco. For the past month his condition had been steadily growing worse. When first taken with dimness and glimmering of sight had severe pains through temples. Can see better on a cloudy day. When it begins to grow dark can see nearly as well as ever. If sees a team way off, coming toward him, it looks like two (Bell., Gels., Nit. ac., Olnd.). After eating, belching of wind; tongue cracked; hands trembled—in fact, seemed to be in a tremor all over; pulse thin and weak, 70; face of a dirty hue; said he had been under many doctors and had taken bucketfuls of drugs, which had not helped him, indeed, medicines did not seem to affect him. While in my office I exhibited a few pellets of Nux v.$^{200}$ (D). The man expressed the utmost surprise and disappointment at receiving a few sugar *parvules*—our allopathic *confreres* use *parvules* now—each about the size of a pin's head. He afterward stated that but for his respect for me he would have ridiculed the idea of such medicine having any effect upon him.

Mr. S. returned to my office in about half an hour, the utmost pleasure and surprise beaming in his face. His hands no longer shook. He eagerly asked me to feel his pulse, which was steadier and fuller. I saw him some days afterward and he assured me he had been better ever since he had taken the small pellets.

